# 145 Co-producing a physical activity referral scheme for the German healthcare system: A qualitative analysis of stakeholder experiences

**DOI:** 10.1093/eurpub/ckae114.050

**Published:** 2024-09-26

**Authors:** Sarah Klamroth, Eriselda Mino, Anja Weissenfels, Inga Naber, Wolfgang Geidl, Peter Gelius, Karim Abu-Omar, Klaus Pfeifer

**Affiliations:** Friedrich-Alexander-Universität Erlangen-Nürnberg, Germany; Friedrich-Alexander-Universität Erlangen-Nürnberg, Germany; Friedrich-Alexander-Universität Erlangen-Nürnberg, Germany; Friedrich-Alexander-Universität Erlangen-Nürnberg, Germany; Friedrich-Alexander-Universität Erlangen-Nürnberg, Germany; Université de Lausanne, Switzerland; Friedrich-Alexander-Universität Erlangen-Nürnberg, Germany

## Abstract

**Purpose:**

Physical activity referral schemes (PARS) are a promising strategy to promote physical activity within healthcare settings. Despite the perceived potential of PARS, no national scheme has yet been implemented in Germany. This study investigated stakeholders’ experiences with the development of a PARS in the German healthcare system using a co-production approach. The focus was on examining facilitators and challenges, along with gathering insights on potential modifications to the joint development process, all from the viewpoint of stakeholders.

**Methods:**

Based on 25 stakeholders participating in the co-production process, seven stakeholders were purposefully selected for one-to-one semi-structured interviews. Following the maximum variation principle, interview partners represented a variety of participating stakeholders regarding the health sector they belong to, the stakeholders’ position within their organization and their attendance during the development phase. We analyzed the interview transcripts using summarizing qualitative content analysis (software MAXQDA 2022).

**Results:**

The following factors were identified as facilitators of the development process: co-production approach, process of co-production, multi-sector stakeholder group, possibility of active participation, coordinating role of researchers, communication, atmosphere and interaction. In contrast, differences in roles and hierarchy, merging of different perspectives, clarification of intervention costs, and competition and conflicting interests were pointed out as challenges. Interviewees made a few suggestions for improving group composition and cooperation among stakeholders.

**Conclusions:**

Overall, stakeholder experiences with the development process were overwhelmingly positive, indicating that co-production is a beneficial approach for the development of PARS intended for integration into healthcare systems. To further improve co-production of PARS models and other interdisciplinary health services, scientists should report on and evaluate the collaborative development processes more frequently and systematically.

**Support/Funding Source:**

The study is funded by the Federal Ministry of Health based on a resolution of the German ‘Bundestag’ by the federal government.

